# Telemedicine-Enabled Medication Optimization for Adults with Polypharmacy: A Systematic Review and Implications for Saudi Vision 2030 Healthcare Transformation

**DOI:** 10.3390/clinpract16100187

**Published:** 2026-10-09

**Authors:** Bobakr Bawazir, Yaslam Ba Srada, Saad Mohammad Aghi, Mohamed Elsayed, Hisham Yousif, Mohamed Ibrahim, Kareem Eltohamy, Mark Sim

**Affiliations:** Medical Engagement Department, Health Horizon Medical Co., Jeddah 21323, Saudi Arabia; yaslam.basardah@bupa.com.sa (Y.B.S.); saad.aghi@bupa.com.sa (S.M.A.); mohammed.alsayedali@bupa.com.sa (M.E.); hisham.yousef@bupa.com.sa (H.Y.); mohammed.ibrahim@bupa.com.sa (M.I.); kareem.eltohamy@bupa.com.sa (K.E.); mark.sim@bupa.com.sa (M.S.)

**Keywords:** telemedicine, telehealth, polypharmacy, medication optimization, medication reconciliation, deprescribing, clinical pharmacy, medication therapy management, digital health, Saudi Vision 2030

## Abstract

Background/Objectives: Polypharmacy may increase the risk of medication discrepancies, drug–drug interactions, potentially inappropriate prescribing, nonadherence, adverse drug events, and avoidable healthcare use. Telemedicine may improve access to medication review, particularly for older adults, people with multimorbidity, and geographically underserved populations. This review evaluated the effectiveness, safety, acceptability, and implementation requirements of telemedicine-enabled medication optimization and considered implications for Saudi Vision 2030. Methods: This PRISMA 2020 systematic review searched PubMed/MEDLINE, Europe PMC, and ClinicalTrials.gov from inception to 30 June 2026. Eligible studies evaluated telemedicine-supported medication reconciliation, medication therapy management, deprescribing, adherence support, or clinical-pharmacy consultation in adults with polypharmacy or explicitly defined high medication risk. Two reviewers independently screened records and extracted data. Clinical and methodological heterogeneity required narrative synthesis. Results: The search identified 168 records; 52 duplicates were removed, 12 reports underwent full-text assessment, and eight studies were included. Green et al. randomized 69 patient-care-partner dyads to immediate or delayed pharmacist telehealth deprescribing; approximately 80% in each arm completed the intervention, but changes in total medication count and regimen complexity were small and the latter was not clinically meaningful. Cole et al. reported 30-day readmission in 12/76 pharmacist-contacted patients and 3/12 patients who declined or could not be reached (*p* = 0.529); this nonrandomized comparison does not establish a reduction in readmissions. Process outcomes, including identification of discrepancies, interactions, and potentially inappropriate medicines, were more consistently favorable than clinical outcomes. Certainty was low for detection of medication-related problems and very low or absent for most clinical, utilization, and economic outcomes. Conclusions: Telemedicine-enabled medication optimization appears feasible, but evidence of clinical effectiveness and cost savings remains uncertain. Saudi implementation should begin with monitored pilots emphasizing safety, equity, Arabic-language accessibility, governance, and prospective evaluation. Registration: This completed review was retrospectively registered on the Open Science Framework (OSF).

## 1. Introduction

Polypharmacy, commonly defined as the concurrent use of five or more medicines, is increasingly prevalent as populations age and multimorbidity becomes more common [1,2]. Multiple medicines may be clinically appropriate, but increasing regimen complexity can raise the risk of potentially inappropriate prescribing, drug–drug interactions, adverse drug reactions, nonadherence, treatment burden, falls, hospitalization, and avoidable healthcare expenditure [2,3]. The World Health Organization identifies medication safety in polypharmacy as a major patient-safety challenge and emphasizes systematic medication review, shared decision-making, and multidisciplinary care [1]. Medication optimization is a patient-centred process intended to ensure that each medicine is evidence-based, clinically appropriate, safe, acceptable, and aligned with the patient’s goals [1,4]. Face-to-face review may be limited by travel, fragmented records, workforce shortages, limited access to pharmacists or specialists, and inadequate follow-up after transitions of care. These barriers may be especially important for older adults, people with disabilities, and those living in rural or underserved communities [3,4]. Telemedicine may help address some of these barriers [5,6]. Videoconferencing, telephone visits, remote monitoring, mobile applications, electronic prescribing, clinical decision-support systems, and shared records can connect patients with pharmacists, physicians, nurses, and multidisciplinary teams. Such models can support medication review, post-discharge reconciliation, identification of prescribing problems, adherence support, therapeutic monitoring, and professional communication [5,6]. However, technology alone does not ensure safer prescribing; effective delivery depends on governance, trained staff, reliable information exchange, prescriber engagement, and patient participation. Remote care can also introduce digital exclusion, limited physical assessment, privacy concerns, unclear professional responsibilities, and uncertain clinical benefit [6]. Existing reviews of telepharmacy and deprescribing have often combined populations without requiring polypharmacy, focused on a single professional service, or emphasized feasibility rather than linking medication, clinical, utilization, economic, and implementation outcomes. The present review therefore focuses specifically on active telemedicine-enabled medication optimization for adults with polypharmacy or defined high medication risk and distinguishes process outcomes from demonstrated clinical effectiveness. This distinction is relevant to Saudi Arabia, where the Health Sector Transformation Program seeks accessible, integrated, person-centred, efficient, and digitally enabled care [7]. Saudi e-health infrastructure and virtual-care services may extend medication expertise across large geographic areas, but international findings require cautious adaptation to local patterns of multimorbidity, health literacy, workforce capacity, digital access, governance, and care pathways [7,8]. The objective was to synthesize medication-related, clinical, patient-reported, utilization, economic, and implementation outcomes of telemedicine-enabled medication optimization and to identify evidence-informed, testable priorities for monitored Saudi pilot programs.

## 2. Materials and Methods

### 2.1. Study Design and Reporting

This systematic review was conducted and reported in accordance with the Preferred Reporting Items for Systematic Reviews and Meta-Analyses (PRISMA) 2020 statement [9]. The review question followed the Population, Intervention, Comparator, Outcomes, and Study Design framework. No protocol was prospectively registered. The completed review was retrospectively registered on the Open Science Framework (OSF; https://doi.org/10.17605/OSF.IO/VZEFR); registration after completion must not be interpreted as prospective preregistration. During full-text assessment, two post hoc clarifications were documented: reports in languages other than English or Arabic could be included when a reliable English abstract and a translatable full text were available, and services using an explicit high-medication-risk criterion could be included when a numerical polypharmacy proportion was not reported. These clarifications did not add a new outcome or change the synthesis method. Because the review analyzed published or publicly available information, institutional ethical approval and informed consent were not required. The completed PRISMA 2020 checklist is cited as Supplementary File S1.

### 2.2. Eligibility Criteria

Studies were eligible when they included adults aged 18 years or older with polypharmacy, as defined by the investigators or, when no definition was provided, concurrent use of at least five prescribed medicines. Mixed-population studies were included when eligible adult data were reported separately or at least 80% of participants met the criterion. A documented post hoc clarification also permitted studies that explicitly enrolled a high-medication-risk population for an active medication-optimization service, even when the proportion using five or more medicines was not reported; these cases are interpreted cautiously. Eligible interventions used synchronous or asynchronous telemedicine to provide active medication optimization, including video consultation, telephone review, telepharmacy, remote reconciliation, adherence monitoring with professional feedback, or multidisciplinary virtual review. Medication optimization required clinical assessment and an actionable recommendation. Comparators included usual care, face-to-face review, another digital intervention, delayed intervention, pre-intervention practice, or no concurrent comparator. Eligible designs included randomized trials, comparative nonrandomized studies, controlled before-and-after studies, interrupted time-series studies, and prospective or retrospective cohorts or service evaluations reporting patient-level or implementation outcomes. Case reports, small case series, protocols, editorials, narrative reviews, qualitative-only studies, cross-sectional surveys without outcome evaluation, and conference abstracts with insufficient data were excluded.

### 2.3. Information Sources and Search Strategy

PubMed/MEDLINE, Europe PMC, and ClinicalTrials.gov were searched from inception to 30 June 2026. As shown verbatim in Appendix A, the PubMed/MEDLINE and Europe PMC strategies operationalized two mandatory concept blocks: (1) polypharmacy or multiple medicines and (2) telemedicine, telehealth, telepharmacy, virtual care, remote consultation, videoconferencing, or mobile health. Medication review and optimization defined intervention eligibility during screening but were not imposed as a third mandatory block because doing so could exclude telemedicine reports that described the clinical activity only in the full text. ClinicalTrials.gov additionally used “medication review” as a search term. No geographic or database language filter was applied. Reports in English or Arabic were assessed directly; the Spanish-language Ponce et al. report was assessed using its English abstract and a translated full text. Reference lists of included reports and relevant reviews were examined; this process did not identify additional eligible records. Records were matched using PMID, DOI, trial-registration number, and normalized title. Registry records were screened as records, linked to publications where applicable, and were not counted as separate included studies. Figure 1 presents the source-specific yields and study-selection counts. No automation tool made eligibility decisions. A source-yield summary and the corresponding search strategies are designated Supplementary Table S1; the original database exports and screening records must be supplied separately for independent verification.

### 2.4. Study Selection

Search results were exported to reference-management software. Automated matching followed by manual verification removed duplicate records. Two reviewers independently screened titles and abstracts against predefined criteria and then assessed potentially eligible full texts. Disagreements were resolved by discussion or third-reviewer adjudication. Reasons for full-text exclusion were recorded. Four reports were excluded after full-text assessment: two qualitative-only studies, one protocol without outcome data, and one cross-sectional report without eligible outcome evaluation. Multiple reports from one investigation were linked and treated as a single study, with the most comprehensive report designated as the primary source. The source-specific search log, screening log, and full-text exclusion log should accompany the submission as supplementary files. The search and selection documentation is cross-referenced in Supplementary Table S1, with underlying record-level logs pending attachment.

### 2.5. Data Extraction

Two reviewers independently extracted data using a standardized, piloted form. Extracted variables included country, setting, design, sample size, participant characteristics, comorbidities, polypharmacy definition, baseline medication burden, intervention technology, participating professionals, intervention intensity, comparator, follow-up, implementation characteristics, and funding. Outcomes included medication appropriateness and count, potentially inappropriate medicines, medication-related problems, discrepancies, adherence, adverse drug events, falls, hospitalization, emergency visits, mortality, health-related quality of life, satisfaction, uptake, digital equity, and costs. Discrepancies were resolved through consensus or third-reviewer arbitration. The complete set of extraction fields is provided in Appendix B.

### 2.6. Risk of Bias Assessment

The only randomized study was assessed with the Cochrane Risk of Bias 2 (RoB 2) tool [10], whereas the seven nonrandomized studies were assessed using the Risk Of Bias In Non-randomized Studies of Interventions (ROBINS-I) framework [11]. For uncontrolled pilot and service-evaluation studies, ROBINS-I domains were used to identify threats to causal interpretation, but the descriptive findings were not treated as comparative effect estimates. Two reviewers made independent domain-level judgments and resolved differences through discussion. Appendix C reports the tool used, domain judgments, overall assessment, and supporting rationale for each study. These judgments influenced the synthesis by giving greater interpretive weight to comparative findings while treating uncontrolled process outcomes as feasibility evidence. See also Table A1 in Appendix C.

### 2.7. Data Synthesis and Statistical Analysis

Study characteristics and findings were grouped by intervention type, care setting, and outcome domain. Meta-analysis was considered only when at least three studies were sufficiently comparable in design, population, intervention, and outcome. This condition was not met, so no pooled estimate was calculated. Narrative synthesis followed SWiM guidance [12] and considered effect direction, consistency, design, and risk of bias. The evidence base was too small and heterogeneous for valid subgroup analyses, sensitivity analyses, funnel plots, meta-regression, or publication-bias tests.

### 2.8. Certainty of Evidence and Implementation Assessment

Certainty was assessed by outcome domain using GRADE considerations [13]: risk of bias, inconsistency, indirectness, imprecision, and publication bias. Because outcomes, designs, and reporting were heterogeneous, certainty was not inferred from direction of effect alone. Appendix D identifies the contributing studies, approximate analytic units where reported, domain-level reasons for downgrading, and final certainty. “No usable evidence” is reported separately from “very-low-certainty evidence.” Implications for Saudi Vision 2030 were developed by the review authors from three explicitly separated inputs: findings of the included studies, contextual information from Saudi policy sources [7,8], and implementation judgments requiring prospective evaluation. See also Table A2 in Appendix D.

## 3. Results

### 3.1. Search Results and Study Selection

The database and registry searches identified 168 records: 99 from PubMed/MEDLINE, 59 from Europe PMC, and 10 from ClinicalTrials.gov. After 52 duplicates were removed, 116 unique records underwent title, abstract, or registry screening. Of these, 104 were excluded because they lacked an eligible adult population, active medication-optimization component, eligible publication type, or relevant outcome. Reference-list screening identified no additional eligible records. Twelve reports underwent full-text assessment. Four were excluded: two qualitative-only studies, one protocol without outcome data, and one cross-sectional report without eligible outcome evaluation. Registry entries were linked to associated publications and were not counted as separate studies. Eight studies were included in the narrative synthesis (Figure 1).

### 3.2. Characteristics of Included Studies

The eight studies, published between 2019 and 2026, represented community care, primary care, nursing homes, ambulatory oncology, hospital clinical-pharmacy consultation, and post-discharge transitional care (Table 1). Designs included one randomized immediate-versus-delayed pilot (69 patient-care-partner dyads) and seven nonrandomized pilot, before-and-after, implementation, prospective single-arm, or retrospective evaluations. Units of analysis included participants, dyads, residents, clinical records, and consultations and therefore were not combined as though they represented equivalent participant samples. Interventions included pharmacist-led video or telephone medication-therapy management, asynchronous specialist consultation, multidisciplinary telegeriatrics, and post-discharge telehealth follow-up. Sample size, eligibility basis, comparator, follow-up, and reporting limitations are shown at study level in Table 1 and Table 2.

### 3.3. Medication-Related Outcomes

Telemedicine interventions consistently generated process outputs, including identification of medication discrepancies, drug–drug interactions, potentially inappropriate medicines, adherence problems, and deprescribing opportunities. These findings should not be equated with demonstrated reductions in medication burden or clinical events. Green et al. randomized 69 patient-care-partner dyads to immediate or delayed pharmacist telehealth deprescribing; 27/34 (79%) and 28/35 (80%), respectively, completed the intervention. Medication-count changes were small, and the reported change in regimen complexity was not clinically meaningful [19]. Cole et al. identified medication-list discrepancies and medication-related problems, but their readmission comparison was nonrandomized: 12/76 pharmacist-contacted patients versus 3/12 patients who declined or could not be reached were readmitted within 30 days (*p* = 0.529) [21]. The 36.8% relative reduction reported by the study therefore reflects an imprecise, selection-prone comparison and does not establish efficacy. Other studies mainly supported feasibility, recommendation generation, or professional acceptance rather than comparative clinical effectiveness. Table 3 and Appendix D summarize the direction and certainty of evidence.

### 3.4. Clinical, Patient-Reported, and Implementation Outcomes

Evidence for clinical and utilization outcomes was sparse and inconsistent. No included study established a reduction in readmissions, emergency consultations, mortality, falls, adverse drug events, or a clinically meaningful improvement in quality of life with adequate control of confounding. Cost evidence was insufficient. Participants and clinicians often valued flexible access, medication education, reduced travel, and pharmacist communication, but these feasibility and acceptability outcomes should be distinguished from clinical effectiveness. Barriers included limited digital literacy or technology access, scheduling difficulties, incomplete information exchange, reimbursement constraints, workload, and variable prescriber uptake. Pharmacist integration, accurate medication lists, multidisciplinary communication, and planned follow-up were recurring facilitators. Figure 2 summarizes the conceptual pathway for telemedicine-enabled medication optimization. Table 4 and Figure 3 present author-developed proposals for monitored Saudi pilots; they are not outcomes directly demonstrated in Saudi settings. Meta-analysis was not performed because of substantial clinical and methodological heterogeneity.

## 4. Discussion

This review suggests that telemedicine-enabled medication optimization is feasible and can support medication-related processes in adults with polypharmacy or defined high medication risk. Remote reviews identified discrepancies, potentially inappropriate medicines, drug interactions, adherence problems, and deprescribing opportunities [14,15,16,17,18,19,20,21]. However, identification of a problem, acceptance of a recommendation, discontinuation of an individual medicine, and a clinically meaningful reduction in total medication burden are distinct outcomes. The included studies more consistently supported feasibility and process improvement than comparative clinical effectiveness. Telemedicine should therefore be understood as a mode of delivering a clinical pathway, not as an intervention whose effectiveness can be inferred from technology use alone.

The effectiveness of telemedicine appeared to depend largely on the involvement of pharmacists and multidisciplinary teams. Pharmacist-led video consultations enabled structured medication histories, clarification of actual medicine use, assessment of adherence, and identification of clinically important interactions. In several studies, pharmacists communicated recommendations directly to physicians, which improved the likelihood that medication-related problems were addressed [14,17,18,19,20,21]. This is particularly important for patients with multiple chronic conditions, because medication optimization requires clinical interpretation, prioritization of risks, and shared decision-making. Digital platforms alone cannot reliably determine whether a medicine remains appropriate for an individual patient.

The evidence for deprescribing was encouraging but inconsistent. Studies involving older adults with dementia, chronic disease, and polypharmacy reported reductions in medicine burden or potentially inappropriate medication use after telemedicine-supported reviews [15,19,20]. However, the magnitude of benefit varied according to the clinical setting, intervention intensity, prescriber engagement, and duration of follow-up. In nursing-home settings, many residents were found to have potentially inappropriate medicines, but acceptance of specialist recommendations by general practitioners was lower than expected [20]. This indicates that identifying medication problems is not sufficient; successful optimization also requires professional authority, timely communication, agreement among clinicians, and a clear system for monitoring treatment after changes.

Telemedicine may be especially useful during transitions of care. Hospital discharge is a high-risk period because medication lists are frequently incomplete, duplicated, or inconsistent with the patient’s pre-admission treatment. Pharmacist-led transitional-care services can identify discrepancies, clarify instructions, educate patients and caregivers, and communicate changes to primary-care teams [21]. These activities may reduce preventable medication errors and unplanned healthcare use. Nevertheless, the evidence for reductions in readmissions and emergency consultations remains preliminary because many studies were observational, used small samples, or lacked comparable control groups.

The review also highlights the importance of patient-centred care. Patients generally valued the convenience of remote consultations, reduced travel, flexible scheduling, and improved access to pharmacists or specialists. Telemedicine may be particularly beneficial for older adults, patients with limited mobility, individuals living in remote areas, and those requiring frequent medication monitoring. However, digital exclusion remains a major concern. Limited internet access, poor digital literacy, hearing or visual impairment, language barriers, and lack of caregiver support may reduce participation and widen existing health inequalities [14,16,19]. Therefore, telephone-based alternatives, Arabic-language interfaces, caregiver involvement, and user-friendly platforms should be incorporated into implementation planning.

The findings have important implications for Saudi Vision 2030 and the Health Sector Transformation Program. Saudi Arabia is developing integrated digital health services, electronic health records, and value-based models of care. Telemedicine-enabled medication optimization represents a practical intervention that can connect primary care, hospitals, community pharmacies, home-care services, and specialist teams. A Saudi model should prioritize patients at greatest risk, including older adults, individuals taking ten or more medicines, patients recently discharged from hospital, people with dementia or multimorbidity, and those receiving high-risk medicines. Pharmacists should have clearly defined responsibilities for medication reconciliation, medication therapy management, adherence support, deprescribing recommendations, and escalation to physicians [19,20].

Implementation should be gradual and accompanied by measurable quality indicators. These should include the number of discrepancies identified and resolved, potentially inappropriate medicines discontinued or justified, high-risk interactions prevented, prescriber acceptance of recommendations, medication adherence, emergency visits, readmissions, patient satisfaction, equity of access, and healthcare costs. Interoperability is essential because medication optimization is weakened when information is fragmented across hospitals, clinics, pharmacies, and patients’ personal records. Governance arrangements should also address consent, privacy, professional accountability, documentation, cybersecurity, and escalation when physical assessment or laboratory testing is required [21].

Despite favorable feasibility and process findings, the evidence does not support definitive claims that telemedicine reduces mortality, hospitalization, adverse drug events, or long-term healthcare expenditure. Risk of bias was driven by small samples, nonrandomized designs, self-selection, uncontrolled before-and-after comparisons, incomplete outcome reporting, and short follow-up. Green et al. provided randomized pilot evidence but was designed primarily for feasibility and was underpowered for definitive clinical effects [19]. Cole et al.’s readmission comparison was particularly vulnerable to selection and comparability bias because the comparator comprised patients who declined or could not be reached [21]. Clinical and methodological heterogeneity precluded pooling. Future research should use pragmatic randomized or carefully controlled designs, prespecified medication and clinical outcomes, longer follow-up, transparent attrition reporting, and prospective economic evaluation.

Overall, telemedicine-enabled medication optimization appears feasible, clinically relevant, and well aligned with Saudi healthcare transformation. Its greatest value is likely to arise when digital communication is combined with pharmacist expertise, multidisciplinary collaboration, interoperable records, shared decision-making, and planned follow-up. Saudi implementation should therefore focus on safe integration into existing clinical workflows, continuous evaluation, and equitable access rather than technology deployment alone.

### 4.1. Strengths

This review was based on a population-intervention-outcome-based framework and PRISMA methodology. It incorporated evidence from primary care, nursing homes, oncology, geriatrics, and transitional care. Medication-related, clinical, patient-reported, and implementation outcomes were considered in the synthesis. Another strength was the translation of the findings into practical priorities for Saudi Vision 2030 for pharmacist integration, interoperability, equity, governance, and sustainability.

### 4.2. Limitations

This review has important limitations. Searches were limited to PubMed/MEDLINE, Europe PMC, and ClinicalTrials.gov; subscription databases such as Embase, CINAHL, Scopus, and Web of Science were not searched, so relevant studies may have been missed. The strategies intentionally used broad polypharmacy and telemedicine blocks without requiring medication-review terminology, but terminology variation and the limited number of sources may still have reduced sensitivity. The Spanish-language Ponce et al. report required translated full-text assessment. A post hoc eligibility clarification allowed explicitly defined high-medication-risk services when a numerical polypharmacy proportion was not reported, which may have increased indirectness. The included studies varied in populations, units of analysis, intervention components, outcomes, and follow-up. Most were pilot, observational, single-arm, or service-evaluation studies and were vulnerable to confounding and selection bias. Risk-of-bias judgments were constrained by incomplete reporting, and meta-analysis was inappropriate. Finally, no included study directly evaluated the proposed Saudi implementation framework; these recommendations are cautious extrapolations that require prospective testing.

## 5. Conclusions

Telemedicine-enabled medication optimization is feasible and can help clinicians identify medication discrepancies, interactions, potentially inappropriate medicines, adherence problems, and deprescribing opportunities. Evidence for clinically meaningful reductions in overall medication burden, readmissions, adverse events, mortality, or costs remains very uncertain. The current evidence therefore supports monitored, pharmacist-integrated pilot implementation rather than claims of established clinical or economic benefit. Such pilots should use interoperable medication records, defined prescriber accountability, shared decision-making, planned follow-up, and prospective safety, equity, effectiveness, and cost evaluation.

## 6. Recommendations

Saudi healthcare institutions should consider monitored pharmacist-led telemedication pilots for older adults, recently discharged patients, people with multimorbidity, and those taking multiple or high-risk medicines. This is an author-developed implementation proposal based on indirect international evidence and Saudi policy context, not a directly demonstrated Saudi outcome. Pilots should connect interoperable medication lists across hospitals, primary care, community pharmacies, and home-care services; provide Arabic-language interfaces, telephone alternatives, caregiver participation, and digital-literacy support; and prespecify escalation pathways. Evaluation should distinguish process outcomes from clinical effectiveness and should include resolved discrepancies, implemented deprescribing decisions, medication burden, adverse drug events, readmissions, patient experience, equity, and cost-effectiveness before wider scale-up.

## Figures and Tables

**Figure 1:**
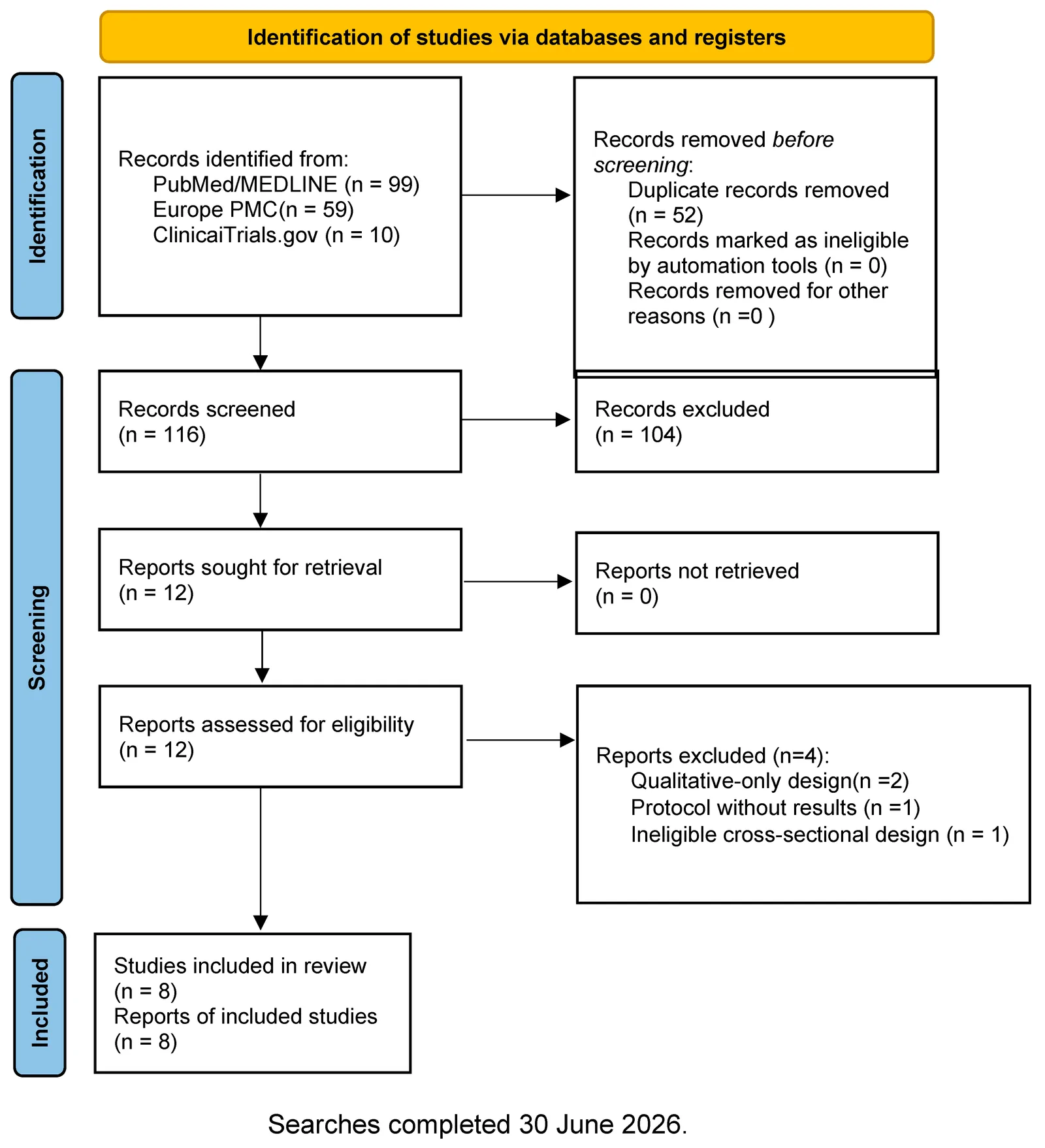
PRISMA 2020 flow diagram for identification, screening, eligibility assessment, and inclusion. Counts reproduce the authors’ reported search log.

**Figure 2:**
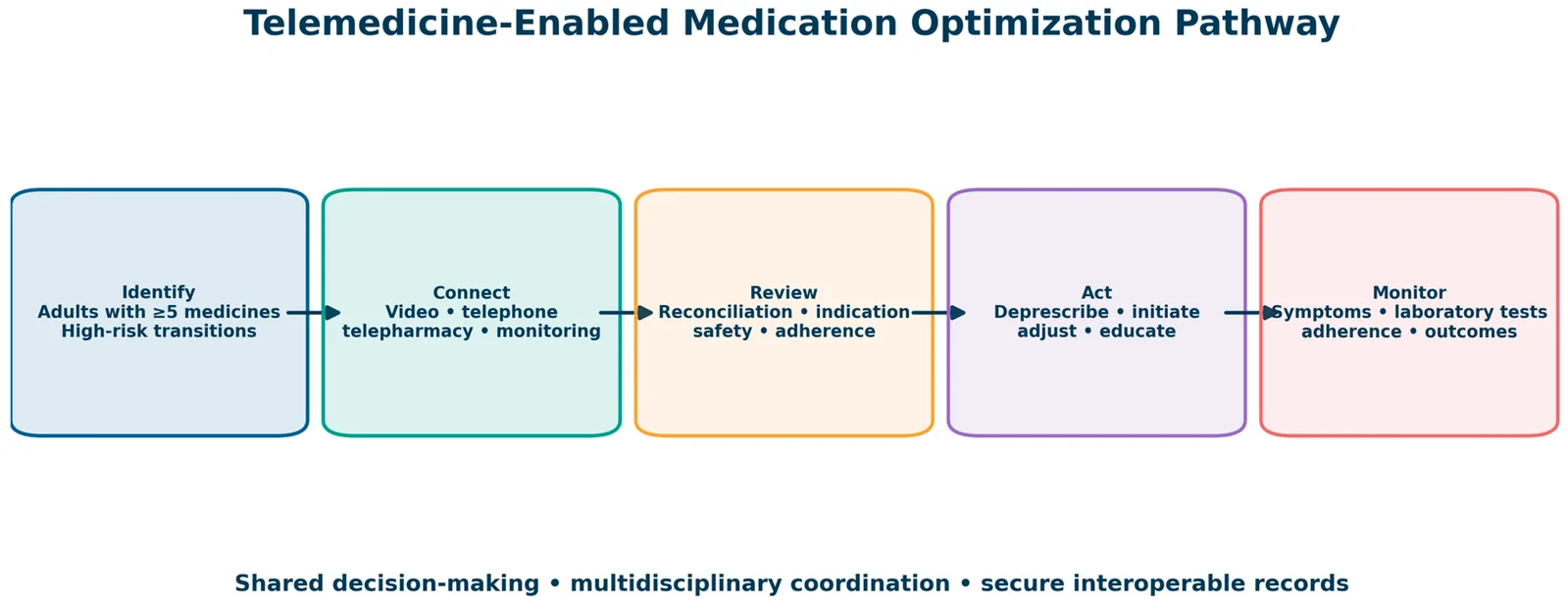
Conceptual pathway for telemedicine-enabled medication optimization in adults with polypharmacy.

**Figure 3:**
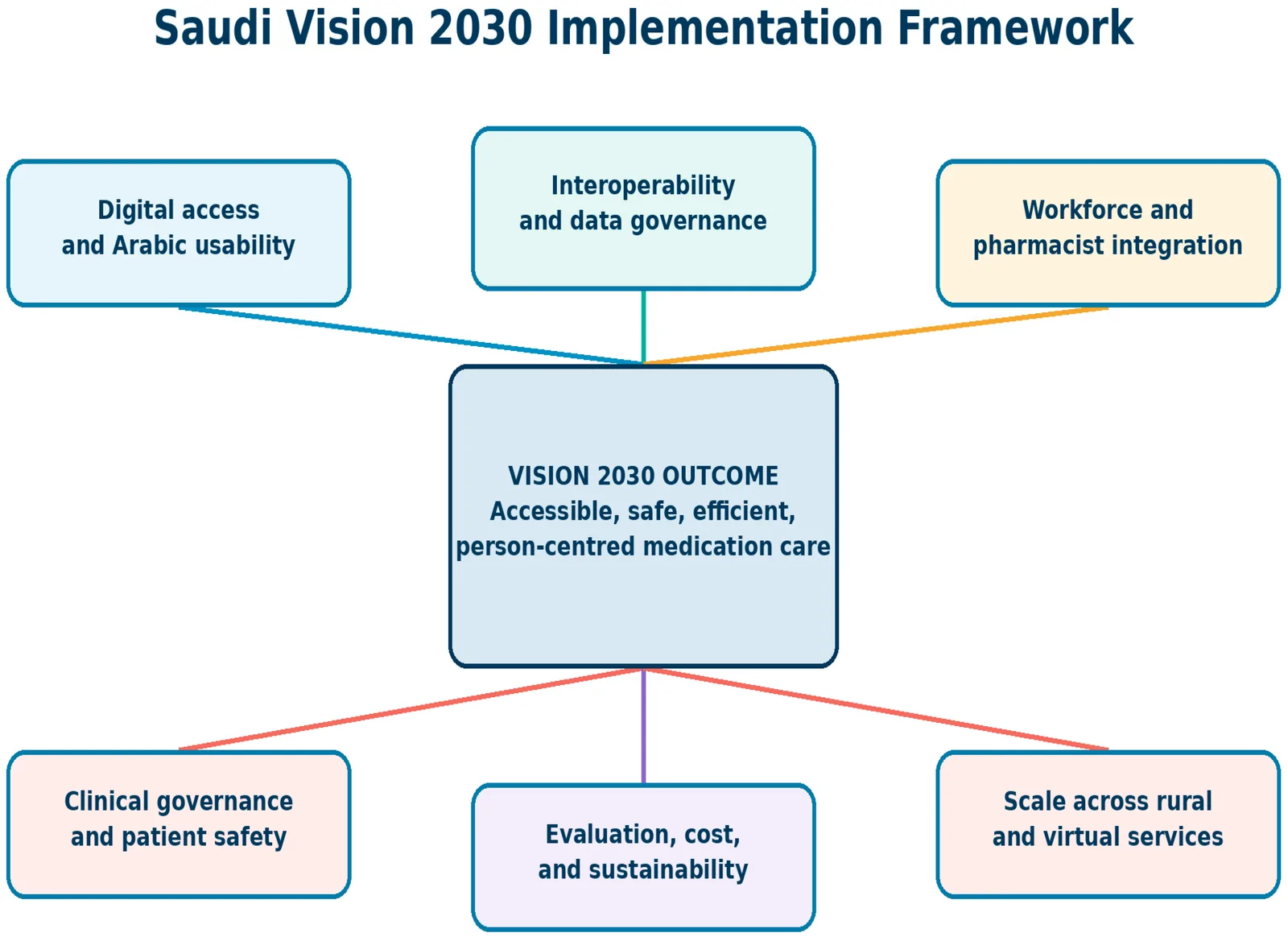
Implementation framework linking medication optimization with Saudi Vision 2030 healthcare transformation.

**Table 1:** Characteristics of the eight included studies.

Study	Design, Comparator, Follow-Up	Population, Sample, and Eligibility Basis	Telemedicine Medication Component
Tyler et al., 2026 [14]	Six-month single-arm pilot; no concurrent comparator	Community-dwelling older adults; ≥65 years and ≥5 medicines; sample size not clearly reported in the review extract	Medication app plus two pharmacist-led virtual MTM sessions
Ponce et al., 2026 [15]	Before-and-after telegeriatrics evaluation; no concurrent control; six-month follow-up	462 asynchronous consultations involving older primary-care patients with investigator-defined polypharmacy; consultation, not unique-patient, denominator	Remote geriatric review with medication-reduction recommendations
Slavova-Boneva et al., 2025 [16]	Implementation/service evaluation; no concurrent comparator; follow-up not clearly reported	Adults aged ≥70 receiving systemic anticancer therapy; numerical polypharmacy proportion not reported; included under high-medication-risk clarification	Remote pharmacist/nurse medicine optimization using the ACCESS pathway
Pehlivanli et al., 2025 [17]	Retrospective single-centre service study; no concurrent comparator	54 clinician-initiated teleconsultations; denominator is consultations, not participants	Electronic clinical-pharmacy consultation and recommendations
Lichtenstein et al., 2025 [18]	Prospective single-arm pilot; no concurrent comparator; one-time consultation with follow-up call	Adults initiating oral anticancer drugs; numerical polypharmacy proportion not reported; included under high-medication-risk clarification	Video medication review focused on drug interactions
Green et al., 2024 [19]	Randomized immediate-versus-delayed pilot; three-month delayed comparator	69 patient-care-partner dyads; people living with dementia using ≥7 medicines; 34 immediate and 35 delayed	Pharmacist-led telehealth deprescribing review
Berard et al., 2022 [20]	Descriptive telemedication service evaluation; no concurrent comparator	39 nursing-home residents in preliminary evaluation; polypharmacy/high medication risk; resident-level denominator	Remote expert multidisciplinary medication review
Cole et al., 2019 [21]	Pilot observational transitional-care evaluation; self-selected/nonreached comparator; 30-day follow-up	76 pharmacist-contacted patients and 12 who declined or could not be reached; included under high-medication-risk clarification	Telephone pharmacist review after discharge

**Table 2:** Telemedicine intervention components and principal reported findings.

Study	Numerical Medication or Process Finding	Clinical, Patient, or Implementation Finding	Interpretive Caution
Tyler et al. [14]	Improved medication knowledge; fewer participants reported forgetting medicines	Virtual MTM participation and technology use increased	Small pilot; self-reported outcomes; no concurrent control
Ponce et al. [15]	462 consultations analyzed; medication count, potentially inappropriate medicines, and unscheduled consultations decreased from baseline; exact effect estimates were not available in the review dataset	Six-month before-and-after telegeriatrics evaluation	Consultations may not equal unique participants; no concurrent comparator
Slavova-Boneva et al. [16]	Medicine optimization and adherence support	Integrated pharmacist/nurse pathway was feasible	Specialist oncology context limits generalizability
Pehlivanli et al. [17]	Interactions, polypharmacy/deprescribing, and adverse reactions commonly addressed	All reported recommendations were accepted; most consults completed within 24 h	Retrospective single-centre process evaluation
Lichtenstein et al. [18]	One-time 30-min pharmacist video consultation identified and addressed oral-anticancer-drug interactions	Feasibility at treatment initiation	Single-arm pilot; numerical effect and uncertainty incompletely reported in the review dataset
Green et al. [19]	69 dyads randomized; 27/34 immediate and 28/35 delayed participants completed the intervention; medication-count changes were small	High completion and feasibility; recommendations frequently accepted	Pilot was not powered for clinical outcomes; regimen-complexity change was not clinically meaningful
Berard et al. [20]	Preliminary evaluation of 39 residents identified 402 potentially inappropriate medicines	Remote expert review feasible in nursing homes	Descriptive uncontrolled evaluation; implementation and prescribing change require confirmation
Cole et al. [21]	12/76 pharmacist-contacted versus 3/12 declined/not reached patients had 30-day readmission (*p* = 0.529); medication discrepancies and problems were identified	Process improvement and care-gap resolution were reported	Comparator was self-selected/nonreached; very small comparator; no established readmission reduction

**Table 3:** Direction and certainty of evidence by outcome domain.

Outcome Domain	Direction of Evidence	Certainty	Contributing Evidence and Reason for Judgment
Detection of medication-related problems	Generally favorable	Low	Eight heterogeneous studies; process findings were recurrent, but seven were nonrandomized and most lacked concurrent controls
Medication knowledge and adherence	Potentially favorable	Very low	Few small studies, mainly self-reported measures, inconsistent definitions, and imprecision
Deprescribing and medication burden	Potentially favorable	Very low	Green et al. (69 dyads) plus uncontrolled studies; small changes and limited clinical meaningfulness
Prescriber acceptance	Favorable in some services	Very low	Service-level process outcome with incomplete denominators and strong context dependence
Readmissions and unscheduled care	Uncertain	Very low	Cole et al. (76 contacted; 12 comparator) and Ponce et al. (462 consultations); serious confounding, unit-of-analysis concerns, and imprecision
Adverse events, falls, mortality, quality of life, and cost	Insufficient evidence	No usable comparative evidence	Outcomes were absent, infrequently reported, or lacked interpretable comparative estimates; a certainty rating was therefore not assigned

**Table 4:** Evidence-informed implementation priorities for Saudi Vision 2030.

Implementation Domain	Recommended Saudi Application	Suggested Evaluation Indicator
Target population	Prioritize older adults, ≥10 medicines, high-risk medicines, multimorbidity, and recent discharge	Eligible patients reached and reviews completed
Workforce	Embed pharmacists within physician-led virtual-care pathways	Recommendations accepted and implemented
Interoperability	Use a reconciled medication list shared across hospitals, primary care, pharmacies, and home care	Discrepancies identified and resolved
Access and equity	Provide Arabic interfaces, telephone alternatives, caregiver participation, and digital-literacy support	Completion by age, region, language, and access group
Safety and governance	Define accountability, consent, documentation, privacy, escalation, and laboratory monitoring	Adverse events, escalations, and follow-up completion
Value and sustainability	Pilot before scale-up and include economic evaluation	Readmissions, emergency visits, patient experience, and cost per resolved problem

## Data Availability

Full search strategies are reproduced in Appendix A and summarized with manuscript-reported source yields in Supplementary Table S1. Study-selection totals and full-text exclusion categories are shown in Figure 1. Underlying database exports, deduplicated screening records, full-text exclusion records, and the completed PRISMA 2020 checklist must be supplied with the supplementary submission or their availability stated accurately before publication. The extraction items are listed in Appendix B. The review was retrospectively registered at https://doi.org/10.17605/OSF.IO/VZEFR.

## Supplementary Materials

The following supporting information can be downloaded at: https://www.mdpi.com/article/10.3390/clinpract16100187/s1, Table S1: Search Strategies and Source Yields; File S1: PRISMA 2020 Checklist.

## Abbreviations

ADE: adverse drug event; EHR: electronic health record; MTM: medication therapy management; PIM: potentially inappropriate medication; PRISMA: Preferred Reporting Items for Systematic Reviews and Meta-Analyses; Saudi Vision 2030: national transformation strategy of the Kingdom of Saudi Arabia; TCM: transitional-care management.

## Appendix A. Search Strategies

### Appendix A.1. PubMed/MEDLINE

(polypharmacy[Title/Abstract] OR polymedication[Title/Abstract]
OR “multiple medication*”[Title/Abstract]
OR “multiple medicine*”[Title/Abstract])
AND
(telemedicine[Title/Abstract] OR telehealth[Title/Abstract]
OR telepharmacy[Title/Abstract] OR “virtual care”[Title/Abstract]
OR “remote consultation*”[Title/Abstract]
OR videoconferenc*[Title/Abstract] OR mHealth[Title/Abstract]
OR “mobile health”[Title/Abstract])
AND
(“1900/01/01”[Date—Publication]: “2026/06/30”[Date—Publication])

### Appendix A.2. Europe PMC

(TITLE_ABS:polypharmacy OR TITLE_ABS: “multiple medications”
OR TITLE_ABS:polymedication)
AND
(TITLE_ABS:telemedicine OR TITLE_ABS:telehealth
OR TITLE_ABS:telepharmacy OR TITLE_ABS: “virtual care”
OR TITLE_ABS:mHealth)
AND
FIRST_PDATE:[1900-01-01 TO 2026-06-30]

### Appendix A.3. ClinicalTrials.Gov

Condition or disease: polypharmacy
Other terms: telemedicine OR telehealth OR telepharmacy
OR virtual care OR medication review

## Appendix B. Data-Extraction Items

The following information was extracted from each eligible study:

1. Author, year, and country
2. Study design and clinical setting
3. Participant characteristics and sample size
4. Definition of polypharmacy
5. Telemedicine modality and intervention duration
6. Professional staff involved
7. Medication-optimization activities
8. Medication-related outcomes
9. Clinical and utilization outcomes
10. Patient-reported outcomes
11. Implementation facilitators and barriers
12. Follow-up duration
13. Limitations and risk-of-bias considerations

## Appendix C. Study-Level Risk-of-Bias Assessments

RoB 2 was applied to Green et al. [19]. ROBINS-I was applied to the remaining nonrandomized studies. Uncontrolled studies were interpreted as descriptive feasibility evidence rather than unbiased estimates of comparative effect.

**Table A1:** Study-level risk-of-bias assessments of the included studies.

Study	Tool	Overall Judgment	Principal Rationale
Tyler et al. [14]	ROBINS-I	Serious	Single-arm pilot; self-reported outcomes; confounding and selection cannot be excluded
Ponce et al. [15]	ROBINS-I	Serious	Uncontrolled before-and-after analysis; consultation-level denominator; secular change and confounding possible
Slavova-Boneva et al. [16]	ROBINS-I	Serious	Service evaluation without concurrent comparator; selective and incomplete numerical reporting
Pehlivanli et al. [17]	ROBINS-I	Serious	Retrospective, single-centre consultation series; selection and outcome-measurement concerns
Lichtenstein et al. [18]	ROBINS-I	Serious	Prospective single-arm pilot; no comparator; short follow-up and imprecision
Green et al. [19]	RoB 2	Some concerns	Randomized delayed-control pilot; approximately 20% did not complete intervention and efficacy reporting was exploratory
Berard et al. [20]	ROBINS-I	Serious	Descriptive uncontrolled evaluation; prescribing uptake and follow-up incompletely reported
Cole et al. [21]	ROBINS-I	Serious	Comparator comprised patients who declined or could not be reached; major selection/confounding and severe imprecision

## Appendix D. Outcome-Level Certainty Assessment

**Table A2:** Outcome-level certainty-of-evidence assessment.

Outcome	Contributing Studies or Units	Final Certainty	Reasons
Detection of medication-related problems	8 studies; mixed participant, dyad, resident, consultation, and record denominators	Low	Downgraded for predominantly nonrandomized designs and indirect process outcomes
Medication knowledge and adherence	Few small studies; exact pooled N unavailable	Very low	Downgraded for risk of bias, inconsistency, indirectness, and imprecision
Deprescribing and medication burden	Green et al., 69 dyads, plus uncontrolled evaluations	Very low	Small or clinically uncertain change; heterogeneous definitions and serious study limitations
Prescriber acceptance	Selected service evaluations	Very low	Context-dependent process outcome with incomplete denominators
Readmissions and unscheduled care	Cole: 76 contacted and 12 comparator; Ponce: 462 consultations	Very low	Serious confounding, non-equivalent groups or units, and imprecision
Adverse events, falls, mortality, quality of life, and cost	No interpretable comparative estimates	No usable evidence	Absence of usable data was not treated as very-low-certainty evidence of no effect

## References

[1] World Health Organization (2019). Medication Safety in Polypharmacy: Technical Report.

[2] Masnoon N., Shakib S., Kalisch-Ellett L., Caughey G.E. (2017). What is polypharmacy? A systematic review of definitions. BMC Geriatr..

[3] Maher R.L., Hanlon J., Hajjar E.R. (2014). Clinical consequences of polypharmacy in elderly. Expert Opin. Drug Saf..

[4] Rankin A., Cadogan C.A., Patterson S.M., Kerse N., Cardwell C.R., Bradley M.C., Ryan C., Hughes C. (2018). Interventions to improve the appropriate use of polypharmacy for older people. Cochrane Database Syst. Rev..

[5] World Health Organization (2021). Global Strategy on Digital Health 2020–2025.

[6] World Health Organization (2022). Consolidated Telemedicine Implementation Guide.

[7] Saudi Vision Health Sector Transformation Program.

[8] Ministry of Health, Kingdom of Saudi Arabia (2024). Digital Transformation and Recovery from COVID-19: National E-Health Strategy.

[9] Page M.J., McKenzie J.E., Bossuyt P.M., Boutron I., Hoffmann T.C., Mulrow C.D., Shamseer L., Tetzlaff J.M., Akl E.A., Brennan S.E. (2021). The PRISMA 2020 statement: An updated guideline for reporting systematic reviews. BMJ.

[10] Sterne J.A.C., Savović J., Page M.J., Elbers R.G., Blencowe N.S., Boutron I., Cates C.J., Cheng H.Y., Corbett M.S., Eldridge S.M. (2019). RoB 2: A revised tool for assessing risk of bias in randomised trials. BMJ.

[11] Sterne J.A., Hernán M.A., Reeves B.C., Savović J., Berkman N.D., Viswanathan M., Henry D., Altman D.G., Ansari M.T., Boutron I. (2016). ROBINS-I: A tool for assessing risk of bias in non-randomised studies of interventions. BMJ.

[12] Campbell M., McKenzie J.E., Sowden A., Katikireddi S.V., Brennan S.E., Ellis S., Hartmann-Boyce J., Ryan R., Shepperd S., Thomas J. (2020). Synthesis without meta-analysis in systematic reviews: Reporting guideline. BMJ.

[13] Guyatt G.H., Oxman A.D., Vist G.E., Kunz R., Falck-Ytter Y., Alonso-Coello P., Schünemann H.J. (2008). GRADE: An emerging consensus on rating quality of evidence and strength of recommendations. BMJ.

[14] Tyler C.C., Armagon A.C., Garcia I.E., Moultry A.M., Yeatts P., Poon I.O. (2026). Perception of technology to support medication management among community-dwelling African American older adults. Sr. Care Pharm..

[15] Ponce S., Galimany J., Fasce G., Arancibia S. (2026). Telegeriatrics for chronic disease management through polypharmacy reduction in primary health care. Rev. Panam. Salud Publica.

[16] Slavova-Boneva V., Barrell A., Battisti N.M.L. (2025). Clinical pharmacist- and clinical nurse specialist-led medicine optimisation and medication adherence using the ACCESS tool. Int. J. Clin. Pharm..

[17] Pehlivanli A., Akyel H., Kadioglu-Yaman B., Basgut B. (2025). The nature of inquiries and responses in clinical pharmacy teleconsultation service: A retrospective study from Türkiye. J. Pharm. Policy Pract..

[18] Lichtenstein M.R.L., Campbell P., Raghunathan R., Beauchemin M., Elkin E.B., Crew K.D., Accordino M., Ippoliti C., Hwang M., Abramova R. (2025). Pharmacist-led video consultation to identify and mitigate drug interactions among patients initiating oral anticancer drugs. JCO Oncol. Pract..

[19] Green A.R., Quiles R., Daddato A.E., Merrey J., Weffald L., Gleason K., Xue Q.L., Swarthout M., Feeser S., Boyd C.M. (2024). Pharmacist-led telehealth deprescribing for people living with dementia and polypharmacy in primary care: A pilot study. J. Am. Geriatr. Soc..

[20] Berard C., Di Mascio T., Montaleytang M., Couderc A.L., Villani P., Honore S., Daumas A., Correard F. (2022). Telemedication reviews to optimize medication prescription for older people in nursing homes. Telemed. J. e-Health.

[21] Cole J., Wilkins N., Moss M., Fu D., Carson P., Xiong L. (2019). Impact of pharmacist involvement on telehealth transitional care management for high medication risk patients. Pharmacy.

