# Supplementary material for: Telemedicine-Enabled Medication Optimization for Adults with Polypharmacy: A Systematic Review and Implications for Saudi Vision 2030 Healthcare Transformation
Source: Clin Pract. 2026 Oct 9;16(10):187. doi: 10.3390/clinpract16100187 (PMC13703827; doi:10.3390/clinpract16100187)
Supplement: Supplementary file 1 [file clinpract-16-00187-s001.zip › File S1 PRISMA_2020_Checklist_Completed.pdf]

PRISMA 2020 Checklist

Review: Telemedicine-Enabled Medication Optimization for Adults with Polypharmacy: A Systematic Review and Implications for Saudi Vision 2030 Healthcare Transformation

Item	Section/Topic	PRISMA 2020 Item	Location in Manuscript
1	Title	Identify the report as a systematic review.	p. 1, title.
2	Abstract	See PRISMA 2020 for Abstracts checklist.	pp. 1–2, structured abstract.
3	Rationale	Describe the rationale for the review in the context of existing knowledge.	pp. 2–3, Introduction.
4	Objectives	Provide an explicit statement of the objective(s) or question(s) the review addresses.	p. 3, final paragraph of Introduction.
5	Eligibility criteria	Specify inclusion and exclusion criteria and how studies were grouped for synthesis.	p. 3, Section 2.2.
6	Information sources	Specify all databases, registers, websites, organisations, reference lists and other sources searched or consulted, and the date each was last searched.	pp. 3–4, Section 2.3; Appendix A.
7	Search strategy	Present the full search strategies for all databases, registers and websites, including filters and limits.	pp. 17–18, Appendix A.
8	Selection process	Specify methods used to decide whether a study met inclusion criteria, including number of reviewers and use of automation.	pp. 4–6, Sections 2.3–2.4.
9	Data collection process	Specify methods used to collect data from reports, including number of reviewers and processes for obtaining/confirming data.	p. 6, Section 2.5.
10a	Data items—outcomes	List and define all outcomes for which data were sought, including whether all compatible results were collected.	p. 6, Section 2.5.
10b	Data items—other variables	List and define other variables for which data were sought and assumptions made about missing/unclear information.	p. 6, Section 2.5; Appendix B.
11	Risk of bias	Specify methods used to assess risk of bias, including tools, reviewers and automation.	p. 6, Section 2.6; p. 19, Appendix C.
12	Effect measures	Specify effect measure(s) for each outcome.	Not applicable to pooled synthesis; no meta-analysis was performed because studies were too heterogeneous. Individual

			numerical results are reported where available.
13a	Synthesis—eligibility	Describe processes used to decide which studies were eligible for each synthesis.	pp. 6–7, Section 2.7.
13b	Synthesis—data preparation	Describe methods required to prepare data for presentation or synthesis.	pp. 6–7, Section 2.7.
13c	Synthesis—tabulation/visual display	Describe methods used to tabulate or visually display results.	pp. 6–7, Section 2.7; Tables 1–4; Figures 1–3.
13d	Synthesis—methods	Describe methods used to synthesize results and rationale for choices.	pp. 6–7, Section 2.7; narrative synthesis following SWiM.
13e	Synthesis—heterogeneity	Describe methods used to explore possible causes of heterogeneity.	p. 6, Section 2.7; subgroup analyses were not conducted because evidence was too small and heterogeneous.
13f	Synthesis—sensitivity	Describe sensitivity analyses conducted to assess robustness.	p. 6, Section 2.7; not conducted because evidence was too small and heterogeneous.
14	Reporting bias assessment	Describe methods used to assess risk of bias due to missing results.	p. 6, Section 2.7; formal publication-bias tests were not performed because the evidence base was too small and heterogeneous.
15	Certainty assessment	Describe methods used to assess certainty/confidence in the body of evidence.	pp. 6–7, Section 2.8; p. 20, Appendix D.
16a	Study selection—results	Describe results of search and selection, ideally with flow diagram.	p. 8, Section 3.1; Figure 1.
16b	Excluded studies	Cite studies that appeared to meet inclusion criteria but were excluded and explain why.	pp. 5 and 8, Sections 2.4 and 3.1; full-text exclusion reasons reported.
17	Study characteristics	Cite each included study and present its characteristics.	pp. 8–10, Section 3.2; Tables 1–2.
18	Risk of bias in studies	Present assessments of risk of bias for each included study.	p. 19, Appendix C, Table A1.
19	Results of individual studies	For outcomes, present summary statistics/effect estimates and precision where available.	pp. 9–11, Tables 2–3 and Section 3.3.
20a	Results of syntheses—contributing studies	Briefly summarise characteristics and risk of bias among studies contributing to each synthesis.	pp. 8–11, Sections 3.2–3.4; Tables 1–3.
20b	Results of statistical syntheses	Present results of all statistical syntheses.	Not applicable; no pooled meta-analysis was performed.
20c	Results of heterogeneity investigations	Present results of investigations of possible causes of heterogeneity.	Narrative discussion in pp. 10–16; formal subgroup/meta-regression analyses were not performed.
20d	Results of sensitivity analyses	Present results of sensitivity analyses.	Not applicable; sensitivity analyses were not performed.
21	Reporting biases	Present assessments of risk of bias due to missing results for each synthesis.	Formal assessment not performed because evidence was too small and heterogeneous; limitation

			stated in Methods/Limitations.
22	Certainty of evidence	Present assessments of certainty/confidence for each outcome.	pp. 10–11, Table 3; p. 20, Appendix D, Table A2.
23a	Discussion—interpretation	Provide a general interpretation in context of other evidence.	pp. 14–16, Discussion.
23b	Discussion—limitations of evidence	Discuss limitations of evidence included in the review.	pp. 15–16, Section 4.2.
23c	Discussion—limitations of review	Discuss limitations of review processes used.	pp. 15–16, Section 4.2.
23d	Discussion—implications	Discuss implications for practice, policy and future research.	pp. 14–17, Discussion, Conclusions and Recommendations.
24a	Registration	Provide registration information, including register name and registration number, or state not registered.	pp. 2–3 and p. 17; retrospective OSF registration DOI 10.17605/OSF.IO/VZEFR.
24b	Protocol	Indicate where protocol can be accessed or state that no protocol was prepared.	p. 3, Section 2.1; no protocol was prospectively registered.
24c	Amendments	Describe and explain amendments to information provided at registration/protocol.	p. 3, Section 2.1; two post hoc eligibility clarifications are described.
25	Support	Describe sources of financial/non-financial support and role of funders/sponsors.	p. 17, Funding; no external funding.
26	Competing interests	Declare any competing interests.	p. 17, Conflicts of Interest.
27	Availability of data/materials/code	Report which materials are publicly available and where.	p. 17, Data Availability Statement; Appendix A; OSF registration.
