# Supplementary material for: Telemedicine-Enabled Medication Optimization for Adults with Polypharmacy: A Systematic Review and Implications for Saudi Vision 2030 Healthcare Transformation
Source: Clin Pract. 2026 Oct 9;16(10):187. doi: 10.3390/clinpract16100187 (PMC13703827; doi:10.3390/clinpract16100187)
Supplement: Supplementary file 1 [file clinpract-16-00187-s001.zip › Supplementary_Table_S1_Search_Strategies_and_Source_Yields.pdf]

Supplementary Table S1. Search Strategies and Source Yields

This table transcribes the strategies and record counts reported in the authors' manuscript. Counts have not been independently validated against original database exports. The original RIS/NBIB/CSV files and screening log must be supplied for verification.

Reported source yields

Source	Reported records
PubMed/MEDLINE	99
Europe PMC	59
ClinicalTrials.gov	10
Total identified	168
Duplicates removed	52
Unique records screened	116
Records excluded at screening	104
Full-text reports assessed	12
Excluded full-text reports	4 (2 qualitative-only; 1 protocol; 1 cross-sectional without eligible outcome evaluation)
Included studies	8

Verification requirements

The author team should attach dated original search exports and the source-level retrieval and screening logs; validate the exact strategies, source yields, overlap, duplicates, excluded records, and trial-publication linkage. This descriptive table is not a substitute for audit-ready search documentation.
